# Notices for September

**Published:** 1861-09

**Authors:** 


### NOTICES FOR SEPTEMBER.

As the proof-reader was nodding, will the reader please wake up, and correct the nonsense, and read on first page, fifth line from the bottom, "arrested"?

Missing.—Subscribers who have failed to receive any number by mail, from January to date, will have the same supplied without charge, by notification. We sometimes miss our most valued exchanges, for weeks and months at a time, such as the *Country Gentleman*, at Albany; which, by the way, does not look as much like a gentleman as it used to; the coat does not look so neat and prim as formerly; it has become dingy. The favorite agricultural weekly of the times deserves a better dress. In eighteen months we have received about half-a-dozen of *Godey's Lady's Book.* Mr. Godey assured us "he didn't do it a purpose," that it was certainly and regularly mailed. The inferences are, that valuable publications are appropriated between the publisher and those who have paid for them.

*Danville Quarterly Review*, published for $3 a year, by Richard H. Collins, 25 West Fourth street, Cincinnati, Ohio, ought to be sustained, not only by the Old School Presbyterian Church, but by the whole Presbyterian family of whatever name. Robert J. Breckinridge, the John Knox of modern Presbytery, is a permanent contributor.

Thomas Jefferson, his Home-Life, by President H. W. Pierson, D.D., is in preparation, the materials having been obtained from a gentleman of position and great wealth, now living, who spent twenty years of his life, including the presidential terms, in the family of Mr. Jefferson, as his confidential adviser and business-agent. These materials are wholly new, and will give a view of the inner family life of the distinguished statesman, which all who admire him will greatly desire to see.

Letter-Paper.—A new kind; one unfolded half-sheet, being at once an economy of fifty per cent to those writing short letters, and avoiding the inconvenience of short lines and the numerous foldings and creases of note-paper. Address, "Mount Holly Paper Company," at Mount Holly Springs, Pennsylvania. Besides its economy and convenience, the quality of the paper and its beauty of finish are not surpassed.

A Sermon, "Church of the Living God," a discourse delivered at Syracuse, New-York, July 16th, 1861, to the General Assembly of the Presbyterian Church, (N. S.,) by its Moderator, Rev. Thornton A. Mills, D.D., of Kentucky. We have not, in a lifetime, read a sermon which surpasses this in the depth of its thoughts, in the grandeur of its ideas, in the comprehensiveness of its suggestions, and the power of its enforcements; the whole pervaded by a spirit of unostentatiousness, earnestness, and piety, well worthy to be a model for all pulpit efforts.

THE SOUTH.—We have no credit subscribers. All subscriptions are paid for up to December, and end with that number. We will carefully preserve the numbers belonging to our Southern friends, and will forward them as soon as peace is established in honor and right. God speed the hour when we all shall become brethren in feelings, in interests, in aspirations, by mutual sacrifices, by the mutual exercise of just, liberal, and lofty views in the light of the present, and of an eternal future; such views as will insure national harmony for all time to come, and the permanent possession of the blessings which belong to that "nation whose God is the Lord."

WE commend to our readers, the patronage of a valuable monthly on health, *Lewis's New Gymnastics*, $1 a year, 20 Essex street, Boston, Mass. It is the next best thing to HALL'S JOURNAL OF HEALTH, published for $1 a year, at 42 Irving Place, New-York.

NOTICE TO EXCHANGES.—Of the budget of exchanges received yesterday, six of the very best of them, as it so happened, had extracts from our JOURNAL without any credit whatever, from a paragraph, up to two columns in length, to wit: *True Union*, of Baltimore, *The Country Gentleman*, of Albany, N. Y., *The Christian Times*, of Chicago, *Lutheran Observer*, of Baltimore, etc. It won't take much time or space, *brudern*, to say: "From HALL'S JOURNAL OF HEALTH." We preserve and circulate, and have for years, all our agricultural and religious exchanges, and at some cost and trouble, with a view to do good and make them known.

ADVICE.—Persons who write for a written opinion of their case, can have it for five dollars, and one general letter of advice for five dollars in addition, the amount to accompany each letter.

